# Musical Sophistication and Speech Auditory-Motor Coupling: Easy Tests for Quick Answers

**DOI:** 10.3389/fnins.2021.764342

**Published:** 2022-01-04

**Authors:** Johanna M. Rimmele, Pius Kern, Christina Lubinus, Klaus Frieler, David Poeppel, M. Florencia Assaneo

**Affiliations:** ^1^Department of Neuroscience, Max-Planck-Institute for Empirical Aesthetics, Frankfurt, Germany; ^2^Max Planck NYU Center for Language, Music and Emotion, New York, NY, United States; ^3^Department of Psychology, New York University, New York, NY, United States; ^4^Ernst Strüngmann Institute for Neuroscience, Frankfurt, Germany; ^5^Instituto de Neurobiología, Universidad Nacional Autónoma de México, Querétaro, México

**Keywords:** speech, auditory-motor coupling, musical sophistication, synchronization, musical training

## Abstract

Musical training enhances auditory-motor cortex coupling, which in turn facilitates music and speech perception. How tightly the temporal processing of music and speech are intertwined is a topic of current research. We investigated the relationship between musical sophistication (Goldsmiths Musical Sophistication index, Gold-MSI) and spontaneous speech-to-speech synchronization behavior as an indirect measure of speech auditory-motor cortex coupling strength. In a group of participants (*n* = 196), we tested whether the outcome of the spontaneous speech-to-speech synchronization test (SSS-test) can be inferred from self-reported musical sophistication. Participants were classified as high (HIGHs) or low (LOWs) synchronizers according to the SSS-test. HIGHs scored higher than LOWs on all Gold-MSI subscales (*General Score, Active Engagement, Musical Perception, Musical Training, Singing Skills*), but the *Emotional Attachment* scale. More specifically, compared to a previously reported German-speaking sample, HIGHs overall scored higher and LOWs lower. Compared to an estimated distribution of the English-speaking general population, our sample overall scored lower, with the scores of LOWs significantly differing from the normal distribution, with scores in the ∼30th percentile. While HIGHs more often reported musical training compared to LOWs, the distribution of training instruments did not vary across groups. Importantly, even after the highly correlated subscores of the Gold-MSI were decorrelated, particularly the subscales *Musical Perception and Musical Training* allowed to infer the speech-to-speech synchronization behavior. The differential effects of musical perception and training were observed, with training predicting audio-motor synchronization in both groups, but perception only in the HIGHs. Our findings suggest that speech auditory-motor cortex coupling strength can be inferred from training and perceptual aspects of musical sophistication, suggesting shared mechanisms involved in speech and music perception.

## Introduction

The beneficial effects of musical training on auditory cognition have long been recognized (Zatorre, 2005). Although, the generalizability of musical training to higher cognitive and non-music-related tasks (beyond pitch processing) has been discussed controversially (Moreno and Bidelman, 2014; Ruggles et al., 2014; Carey et al., 2015), many studies report beneficial effects on auditory perception. For example, musical training has been suggested to increase auditory working memory (Zhang et al., 2020), aspects of auditory scene analysis (Pelofi et al., 2017), or inhibitory control (Slater et al., 2018). Furthermore, many studies have reported that musical training affects speech perception in noise (Parbery-Clark et al., 2011; Strait and Kraus, 2011; Swaminathan et al., 2015; Varnet et al., 2015; Zendel et al., 2015; Coffey et al., 2017a; Puschmann et al., 2018; Yoo and Bidelman, 2019), while additional variables might affect the outcome of such a comparison (Ruggles et al., 2014; Boebinger et al., 2015; Yoo and Bidelman, 2019; for a review see, Coffey et al., 2017b). On a neuronal level, a beneficial effect of musical training on speech perception has been related to increased auditory-motor coupling and synchronization (Bailey et al., 2014; Du and Zatorre, 2017; Puschmann et al., 2018). For example, improved syllable perception at varying noise levels in musicians compared to non-musicians was accompanied by increased left inferior frontal and right auditory activity (Du and Zatorre, 2017). Furthermore, the intrahemispheric and interhemispheric connectivity of bilateral auditory and frontal speech motor cortex was enhanced in musicians. The impact of musical training on speech perception through auditory-motor coupling might also be related to working memory improvements due to more efficient sensorimotor integration (Guo et al., 2017).

Musical training has been shown to improve speech perception skills at a behavioral (Parbery-Clark et al., 2011; Swaminathan et al., 2015; Varnet et al., 2015) and neuronal level (Strait and Kraus, 2011; Zendel et al., 2015; Du and Zatorre, 2017; Puschmann et al., 2018, 2021). Furthermore, auditory and motor processing are highly interactive during music and speech perception and production (Hickok and Poeppel, 2007; Zatorre et al., 2007; Hutchins et al., 2014; Assaneo and Poeppel, 2018; Assaneo et al., 2021). In line with these observations, overlapping neural circuitry has been suggested for music and speech, but it is unclear to what extent such an overlap reflects shared processing mechanisms (Peretz et al., 2015; Sammler, 2020; Sammler and Elmer, 2020). For example, the tracking of the (rhythmic) temporal structure has been reported in the auditory cortex for speech (e.g., Luo and Poeppel, 2007; Gross et al., 2013; Hyafil et al., 2015; Rimmele et al., 2015, 2021; Keitel et al., 2018; Kösem et al., 2018) and music (Doelling and Poeppel, 2015; Tal et al., 2017; Harding et al., 2019; Di Liberto et al., 2020). While, at the same time, distinct neural circuitries for music and speech processing have been reported (Norman-Haignere et al., 2015; Chen et al., 2021). Understanding whether the shared mechanisms of music and speech processing exist is crucial, as it opens possibilities to enhance language acquisition and literacy via musical training (Peretz et al., 2015). However, because musicians and non-musicians are often compared, it remains unclear whether such shared mechanisms reflect the effects of musical training or reflect other aspects of musicality (which might not genuinely be due to the training).

Recent research has indicated wide individual differences in auditory-motor interactions during speech production and perception (Assaneo et al., 2019a, 2021; Kern et al., 2021). More precisely, assessing speech-to-speech synchronization in the general population yields a bimodal distribution: while a subgroup (HIGH synchronizers) spontaneously aligns the produced syllabic rate to the perceived one, the rest do not show an interaction between the produced and perceived rhythms (LOW synchronizers). At the brain level, HIGH synchronizers show increased functional and structural coupling between auditory and motor cortices (Assaneo et al., 2019a). Further findings corroborate cognitive differences between the groups (i.e., HIGHs and LOWs). HIGHs performed better on a statistical learning task (Assaneo et al., 2019a), showed improved syllable perception in a noisy environment (Assaneo et al., 2021), and enhanced auditory temporal processing of non-verbal sequences (e.g., rate discrimination; Kern et al., 2021). Although a correlation between group affiliation and years of musical training has been shown (Assaneo et al., 2019a), the extent to which speech auditory-motor coupling and musicality or musical training are related is unknown.

Another relevant question is whether musical training might impact the relationship with auditory-motor coupling differently depending on the type of musical instrument individuals are trained on. Previous research suggests that for example, percussion instruments might particularly train rhythmic motor abilities. These were related to improved inhibitory control and have been shown to more strongly impact speech in noise perception compared to vocal training (Slater and Kraus, 2016; Slater et al., 2017, 2018). However, other studies did not report the effects of the type of musical instrument on the training benefit for auditory psychophysical measures (comparing violinist and pianists: Carey et al., 2015), or on an age-related benefit from musical training for speech perception in noise (comparing several instrument families: Zhang et al., 2020). Both, auditory-motor coupling (Assaneo and Poeppel, 2018; Assaneo et al., 2019a, 2021) and speech perception (Ghitza, 2011; Giraud and Poeppel, 2012; Gross et al., 2013; Hyafil et al., 2015; Rimmele et al., 2015, 2018, 2021; Kösem and van Wassenhove, 2017) have been related to rhythmic processing. Thus, we were interested in whether training on different types of instruments affects the relation between musical sophistication and auditory-motor coupling.

In the past, musicality has been studied in terms of musical training, but a broader conceptualization of musicality beyond training has been proposed (Levitin, 2012; Müllensiefen et al., 2014). The Goldsmiths Musical Sophistication Index (Gold-MSI) (Müllensiefen et al., 2014) differentiates the following aspects of musical sophistication with a focus on the general population (i.e., no professional musicians): *Active Engagement, Perceptual Abilities, Musical Training, Singing Abilities, Emotional Attachment*, and the scale *General Sophistication* (i.e., a score computed based on all subscales, Müllensiefen et al., 2014; GS). It is unknown, which of these aspects of musical sophistication share common mechanisms with speech perception and more specifically with speech auditory-motor coupling. For example, *Musical Training* and *Singing Abilities* might be related to speech perception through their effect on auditory-motor coupling. Perceptual musical abilities might reflect several aspects of auditory perception, which might also affect speech perception and auditory-motor coupling. In contrast, it is unclear whether emotional attachment to music could be related to speech perception.

The present study investigates the relationship between aspects of musical sophistication and speech auditory-motor coupling by using the Gold-MSI self-inventory of musical sophistication (Goldsmiths Musical Sophistication Index; Gold-MSI; Müllensiefen et al., 2014) and a behavioral test that enables the estimation of speech auditory-motor coupling through the spontaneous synchronization between speech perception and production (SSS-test; Assaneo et al., 2019a). We hypothesize that certain aspects of musical sophistication allow us to predict speech auditory-motor synchronization strength. Specifically, the subscore *Musical Training* and to a smaller extend *Perceptual Abilities* were expected to be predictive. In contrast, we did not expect the effects of the subscales *Active Engagement* and *Emotional Attachment*. Furthermore, we explored whether training with certain musical instruments is particularly related to high speech auditory-motor synchronization behavior.

## Materials and Methods

### Participants

Overall 196 healthy participants, recruited from the local Frankfurt/M area in the context of two studies (Assaneo et al., 2021; Kern et al., 2021), were included in the analysis (female = 128; mean age: 24.9, StD: 3.8; 2 participants had been excluded prior to the analysis because of missing values for some of the Gold-MSI subscales). While men are underrepresented in our sample, this should not induce a bias in the results since previous work showed no correlation between gender and being a high or a low synchronizer (Assaneo et al., 2019a). According to the self-report, participants had normal hearing, no neurological or psychiatric disorder, no dyslexia, and were not taking psychotropic substances during the last 6 months. The experimental procedures were ethically approved by the Ethics Council of the Max Planck Society (No. 2017_12).

### Procedure

The present work represents a new set of analyses performed on already published data. The data were collected in the context of two studies that included additional experimental procedures; for more detail of the complete protocols please refer to the original papers by Assaneo et al. (2021) and Kern et al. (2021). The results of the SSS-test were already reported in these studies, but the Gold-MSI data were only reported for one (Kern et al., 2021). The effectiveness of both versions of the SSS-test to split the general population into two groups with significantly different structural and functional brain features, as well as different performance on cognitive tasks, has been reported in previous works and is not part of the current study (Assaneo et al., 2019a, b, 2020, 2021; Kern et al., 2021). Here, we focus on the relationship between group pertinence and general musical abilities.

The individual speech auditory-motor coupling strength was estimated based on two versions of the spontaneous speech synchronization test (SSS-test). Data collected with an implicit version of the test comes from Assaneo et al. (2021), and data corresponding to an explicit version from Kern et al. (2021). Here we briefly describe the test, for more detail please refer to the original studies.

During both versions of the test, participants listened to an 80 s rhythmic train of syllables and were instructed to focus on the syllable sequence while concurrently and continuously whispering the syllable /TE/. Their task was to either synchronize their speech production to the syllable sequence (explicit SSS-test; Kern et al., 2021) or to perform a syllable recognition task at the end of the sequence (implicit SSS-test; Assaneo et al., 2021). The purpose of the syllable recognition task is to direct the participant’s attention to the syllable detection task and to avoid them intentionally synchronizing their whisper to the auditory stimulus. There is no useful information in the participants’ responses, as it has been shown that low and high synchronizers have equally poor performance on this task (Assaneo et al., 2019a). For this reason, participants’ responses were not saved.

For both versions, the auditory stimulus comprised 16 different syllables, not including the syllable/TE/. In the implicit SSS-test, syllables were presented at a rate of 4.5 Hz. In the explicit SSS-test, the random syllable train contained a progressively increasing rate (*M* = 4.5 Hz, range: 4.3–4.7 Hz, steps: 0.1 Hz after 60 syllables). For more detail about the stimulus refer to Assaneo et al. (2019b).

After the experiment, participants filled out the German version of the Goldsmiths Musical Sophistication Index (Gold-MSI) (Müllensiefen et al., 2014; Fiedler and Müllensiefen, 2015) and a demographics questionnaire.

### Analysis

All data analyses were performed in Matlab 2020a (version 9.8) and R (version 4.0.5) and visualized in Matlab 2020a (version 9.8) and Python 3.6.9,^1^ using the libraries Seaborn 0.11.1 (Waskom, 2021) and Matplotlib 3.2.2 (Hunter, 2007).

### Participants Classification as High or Low Synchronizers

**FIGURE 1:**
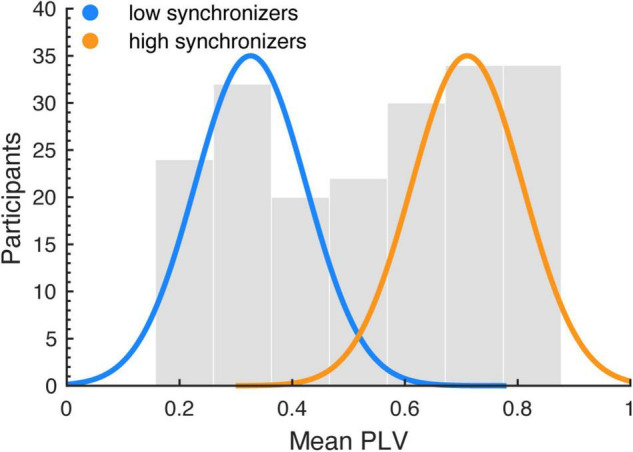
Auditory-motor coupling strength was estimated using the spontaneous speech-motor synchronization test (Assaneo et al., 2019a). The histogram shows the distribution of the mean PLVs (averaged across runs) computed between the speech production and perceived audio signals, for the whole sample (including the samples of both studies). A k-means algorithm was employed to segregate the population into two clusters: low (LOWs) and high (HIGHs) synchronizers. The blue and orange lines indicate normal distributions fitted to each cluster.

Following Assaneo et al. (2019a), speech auditory-motor synchronization (SSS-test) was analyzed by computing the phase-locking value (PLV) (Lachaux et al., 1999) between the envelope of the recorded speech production signal and the cochlear envelope of the presented audio stimulus. It has been suggested that the envelope is a part of speech acoustics particularly relevant for speech perception (Smith et al., 2002). The envelope reflects the slow amplitude modulations present in the broadband acoustic speech signal. The cochlear envelope denotes the average envelope across a range of narrow frequency bands (auditory channels: 180–7,246 Hz), resembling acoustic processing in the cochlea (i.e., the cochlear frequency maps; Yang et al., 1992; Wang and Shamma, 1994). The cochlear envelope of the speech stimulus was computed using the NSL Auditory Model toolbox for Matlab. Next, the envelope was computed for the produced signal (without the cochlear filtering). Then, the phases of the envelopes were extracted using the Hilbert transform (downsampling: 100 Hz; band-pass filtering: 3.5–5.5 Hz). The phases of the perceived (stimulus) and produced signals were used to estimate the PLV in windows of 5 s with an overlap of 2 s (Lachaux et al., 1999). For each run, the mean PLV across windows was assigned as the synchrony measurement. Since previous studies have shown that the synchronization measurement follows a bimodal distribution, a k-means clustering (Arthur and Vassilvitskii, 2007) algorithm with 2 clusters was applied to the mean PLVs across the two experimental blocks to divide the sample (including all participants from both studies reported here) into two groups, high and low synchronizers (HIGHs: *n* = 109; LOWs: *n* = 87; Figure 1). The same procedure was applied in previous research showing a connection between the high and low synchronizer affiliation and auditory-motor cortex coupling strength (Assaneo et al., 2019a, 2021; Kern et al., 2021). Hartigans dip-test (Hartigan and Hartigan, 1985) revealed a trend toward rejecting unimodality (D = 0.037, *p* = 0.056). This replicates previous findings showing bimodality (significant rejection of unimodality) in a larger sample, and a trend toward significance with a sample size comparable to ours (Assaneo et al., 2019a).

### Relationship Between the Goldsmiths Musical Sophistication Index and the Synchronization Test

Based on the 38 items of the Gold-MSI (Müllensiefen et al., 2014; German version: Schaal et al., 2014) the five subscales *Active Engagement, Perceptual Abilities, Musical Training, Singing Abilities, Emotional Attachment*, and the scale *General Sophistication* (GS) were computed.

Group differences in median scores between high and low synchronizers were analyzed using Wilcoxon rank sum tests (two-sided; Bonferroni corrected *p*-value at0.05: *p*_*corr*0.05_ = 0.0083, and at0.01: *p*_*corr*0.01_ = 0.0017). Additionally, the HIGHs and LOWs were tested separately to establish whether the median score for each subscale and the General Sophistication differed from the mean English-speaking norm population reported in Müllensiefen et al. (2014), as well as a previously reported German-speaking sample (Schaal et al., 2014), using Wilcoxon rank sum tests (two-sided: Bonferroni correction, *p*_*corr*0.05_ = 0.0042; *p*_*corr*0_._01_ = 0.0008).

Next, chi-squared tests were used to test for differences in the distribution (within and across groups) of instruments participants reported training on. The instruments were categorized as instrument families: *none, strings, voice, woodwinds, keys, percussion, brass*.

**FIGURE 2:**
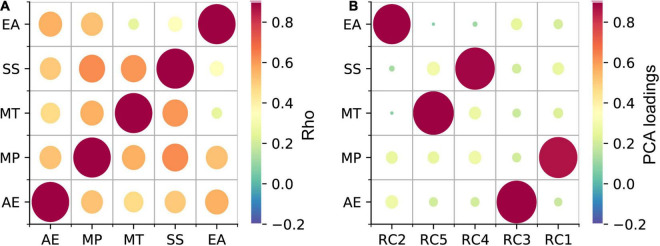
Gold-MSI subscale scores were decorrelated using PCA. **(A)** Strong to moderate correlations between the scores of the Gold-MSI subscales were observed. **(B)** Load of the 5 PCA components on the *Active Engagement* (AE), *Musical Perception* (MP), *Musical Training (MT)*, *Singing Skill (SS)*, and *Emotional Attachment (EA)* subscales of the Gold-MSI. The color and size of the circles indicate the coefficient (rho) of the Spearman correlation and the loading strength in panels A and B, respectively (see scale).

To investigate, whether the high or low synchronizer behavior can be inferred from any of the subscales of the Gold-MSI, a latent class regression model was fitted (Leisch, 2004). One of the assumptions of the analysis that must be met is the absence of multicollinearity. As the scores of the Gold-MSI subscales were moderate to highly correlated (Spearman correlation; rho: 0.25–0.63; Figure 2A), Principal Component Analysis (PCA; varimax rotation; 5 components; variance explained: Component2: 21%, Component5: 21%, Component4: 20%, Component3: 20%, Component1: 19%) was performed to decorrelate the subscales. The projection of the original data on the PCA vector space was retrieved by multiplying the original data with the PCA eigenvectors. In a latent class regression model, two clusters formed based on the PLV values were inferred using the projected data on the five PCA components as predictors (metrical variables: Component1-Component5). The group affiliation of HIGHs and LOWs was used as the initial cluster assignment of observations at the start of the algorithm. Finally, to identify subscales that were relevant for predicting the clusters, the PCA components were related to the Gold-MSI subscales by inspecting the component loadings (i.e., indicating the correlation between the subscales and the PCA components) (Figure 2B).

## Results

**FIGURE 3:**
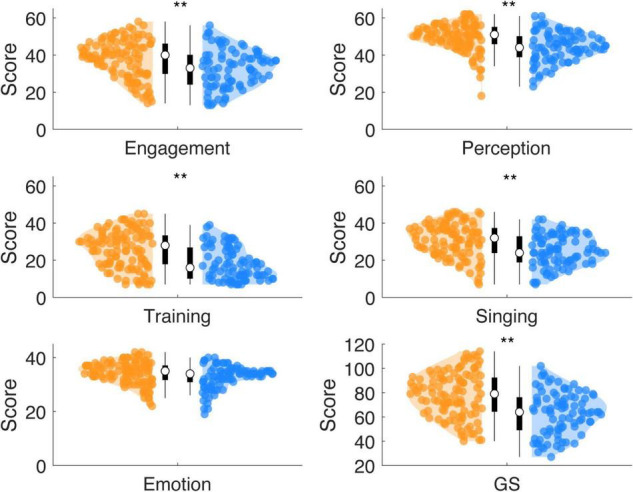
HIGHs show increased scores for most subscales of musical sophistication, while compared against LOWs. The scores (cumulated across items) are displayed for each subscale of the Gold-MSI (Active Engagement, Musical Perception, Musical Training, Singing Abilities, Emotional Attachment) and the General Score (GS). HIGHs showed increased mean scores for all subscales, but the Emotional Attachment scale (**: Bonferroni corrected *p* < 0.01; using Wilcoxon rank sum test).

Wilcoxon rank sum tests showed for high synchronizers increased Gold-MSI scores compared to low synchronizers (Figure 3) for the general factor *GS* (*W* = 12,772, *p* < 0.0001, *r* = 0.261) and the subscales *Active Engagement* (*W* = 12,205, *p* = 0.0002, *r* = 0.188), *Perceptual Skills* (*W* = 12,663, *p* < 0.0001, *r* = 0.246), *Musical Training* (*W* = 1,278, *p* < 0.0002, *r* = 0.261), and *Singing Skills* (*W* = 1,256, *p* < 0.0001, *r* = 0.233). No effects were observed for the subscale *Emotional Attachment* (*W* = 1,172, *p* = 0.0125, *r* = 0.126; Bonferroni corrected *p*_*corr*0_._05_ = 0.0083).

**FIGURE 4:**
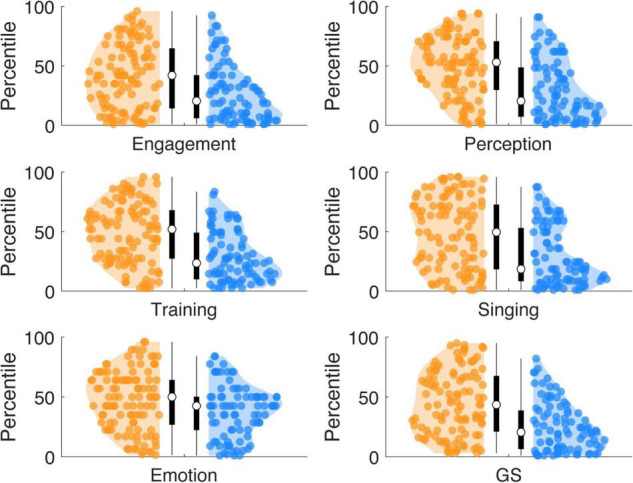
Percentiles of musical sophistication scores with respect to an English-speaking norm population. High synchronizers show a similar distribution compared to an English-speaking norm population, with the median around the ∼50 percentile. In contrast, low synchronizers showed lower scores of musical sophistication at all scales, with the median in the lower percentiles (∼30).

Compared to the originally reported English-speaking norm population (Müllensiefen et al., 2014; Supplementary Table 3; sample: *n* = 147,633; norm population mean scores: *Active Engagement:* 42, Musical *Perception: 50, Musical Training: 27, Singing Skills: 32, Emotional Attachment: 35, General Sophistication: 82*), high synchronizers showed a similar distribution, while low synchronizers showed lower scores (Figure 4). Wilcoxon rank sum tests show that the scores of high synchronizers did differ from the mean of the norm population for the *Active Engagement s*cale (*W* = 2,015; *p* = 0.0030; *r* = −0.201; *p*_*corr*0_._05_ = 0.0042), but not for any of the other subscales (*p*s: 0.9891, 0.5977, 0.7543, 0.6774, 0.0835). In contrast, the scores of low synchronizers differed from the mean of the norm population for the *General Sophistication index* and all subscales (*W*s ∼ [267, 423, 561, 379, 533, 1021], *r*s ∼[−0.529, −0.479, −0.434, −0.493, −0.443, −0.287]) (*p*s < 0.0002; *p*_*corr*0.05_ = 0.0042). Low synchronizers on average scored*: Active Engagement* 33, *Musical Perception* 44, *Musical Training* 18, *Singing Skills* 25, *Emotional Attachment* 33, *General Sophistication* 63. In contrast, high synchronizers on average scored higher: *Active Engagement* 38, *Musical Perception* 50, *Musical Training* 26, *Singing Skills* 31, *Emotional Attachment* 34, *General Sophistication* 78. A comparison of our full sample (high and low synchronizers taken together) to the English norm population showed that our sample scored lower compared to the norm on all scales but the *Emotional Attachment* scale (*Active Engagement, Musical Training, Singing Skills, General Sophistication*, Ws ∼[4,326, 6,266, 5,549, 6,170, 4,651], rs ∼[−0.338, −0.215, −0.261, −0.221, −0.318], *p*s < 0.0042; *p*_*corr*0.05_ = 0.0042)*(Emotional Attachment scale:* W = 7,458, *r* = −0.14, *p* = 0.0057).

We additionally compared our data to a German-speaking sample (Schaal et al., 2014; *n* = 641; Table 1, mean scores: *Active Engagement*: 33, *Musical Perception*:46, *Musical Training*: 23, *Singing Skills*: 28, *Emotional Attachment*: 31, *General Sophistication*: 70), which, however, was much smaller in size compared to the English norm population. High synchronizers scored above the German sample for all subscales and the *General Sophistication* scale (*W*s ∼[4,578, 4,822, 4,046, 4,231, 5,241, 4,319], *r*s ∼[0.324, 0.374, 0.215, 0.253, 0.46, 0.27], *p*s < 0.0016; pcorr0.05 = 0.0042); low synchronizers scored below the norm for the subscale *Musical Training* and the *General Sophistication* scale (*W*s ∼[869, 1135], *r*s ∼[−0.335, −0.25], *p*s < 0.001; pcorr0.05 = 0.0042), and above the norm for the subscale Emotional Attachment (*W* = 2935, *r* = 0.328, *p* < 0.00002). No differences were observed for the other subscales. Our full sample (high and low synchronizers taken together) compared to the German sample, showed no significant difference for the subscales *Musical Training, Singing Skills* and the *General Sophistication* score. In contrast, our sample scored higher on the *Active Engagement*, *Musical Perception* and *Emotional Attachment* scales (*W*s ∼[12,526, 12,318, 15,989], *r*s ∼[0.183,0.169, 0.403], *p*s < 0.0009; pcorr0.05 = 0.0042).

**FIGURE 5:**
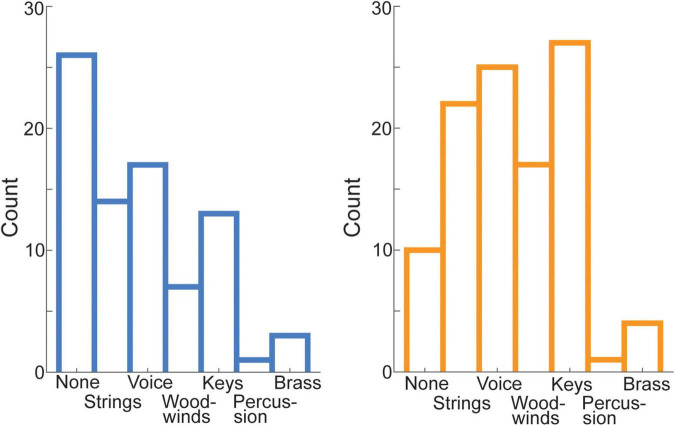
Distribution of the trained instrument across HIGHs and LOWs. HIGHs (displayed in orange) and LOWs (displayed in blue) differed with respect to how many individuals received musical training. However, the distribution of instrument families did not differ, when the category “none” was removed.

Chi-squared tests showed the distribution of instruments participants reported musical training on (for this analysis, several participants had to be excluded because of missing values; *n* = 187) varied across groups [χ*^2^*(6) = 16.58, *p* = 0.011] (Figure 5). However, the effect was related to more HIGHs compared to LOWs reporting that they received training on any instrument (category “none” for HIGHs: 10 and LOWs: 26). If the category “none” was removed, there were no differences in distribution across the groups [χ^2^(5) = 1.49, *p* = 0.914]. The distribution was different from a uniform distribution within groups even when the category “none” was removed, e.g., few participants reported training on percussion while many participants reported training on keys [HIGHs: χ^2^(5) = 38, *p* < 0.001; LOWs: χ^2^(5) = 22.78, *p* < 0.001].

**FIGURE 6:**
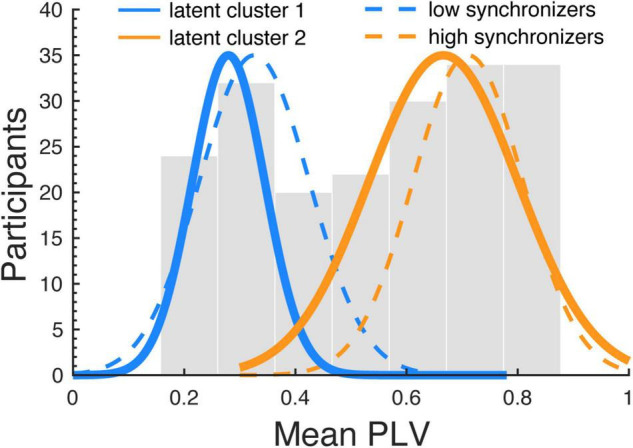
Distribution of the clusters revealed from the latent class regression analysis. The histogram shows the distribution of the mean PLVs (averaged across runs) computed between the speech production and perceived audio signals, for the whole sample (including the samples of both studies). The dashed blue and orange lines indicate normal distributions fitted to the cluster revealed by the k-means algorithm segregating two clusters (HIGHs and LOWs); the thick blue and orange lines indicate the clusters revealed by the latent class regression analysis. The two clusters highly overlap for the different analyses.

In a next step we analyzed to what extend the synchronizer group affiliation (HIGHs, LOWs) can be inferred from the Gold-MSI subscales. After the subscales were decorrelated, the 5 PCA components were used as predictors in a latent class regression model. The latent class regression revealed two clusters that were medium well separated (cluster1: ratio = 0.67, cluster2: ratio = 0.67; ratios indicate the overlap in posterior probability of cluster belonging, optimally separated components show ratios close to 1; Leisch, 2004). With the following centroids and cluster sizes (cluster1, c = 0.28, *n* = 64, cluster 2: c = 0.67, *n* = 132), cluster1 corresponded to the LOWs and cluster2 to the HIGHs as revealed with the k-means algorithm (Figure 6). For cluster1 (describing the LOWs) a significant intercept was observed (β = 0.3; SE = 0.01; *z* = 23.83; *p* < 0.001) and the significant effects of the predictor Component5 (β = 0.03; SE = 0.01; *z* = 3.25; *p* = 0.001). For cluster2 (describing the HIGHs) a significant intercept was observed (β = 0.64; SE = 0.02; *z* = 35.44; *p* < 0.001) and significant effects of the predictors Component1 (β = 0.05; SE = 0.02; *z* = 3.08; *p* = 0.002) and Component5 (β = 0.05; SE = 0.01; *z* = 3.83; *p* < 0.001), as well as a trend for Component4 (β = 0.02; SE = 0.01; *z* = 1.95; *p* = 0.051). The two PCA components that showed significant effects showed the highest loading on the Gold-MSI factors *Perceptual Skills* (Component 1) and *Musical Training* (Component 5) (Figure 2B). The PCA component that showed a trend loaded highest on *Singing Skills* (Component 4). The Component 3, which loaded highest on the subscale *Active Engagement* and Component 2, which loaded highest on the subscale *Emotional Attachment*, showed no significant effect. The analysis was run on the PCA components in order to deal with the multicollinearity between the Gold-MSI subscales. A downside of the PCA based analysis is that it might blur the interpretability compared to the original subscales For interpretation purposes, we related the PCA components to the Gold-MSI subscale with the highest loading. Thus, as a control we ran the same analysis on the original Gold-MSI subscales data, which are medium to highly correlated. The analysis widely confirms the findings of our PCA based analysis. The latent class regression again revealed two clusters that were medium well separated (cluster1: ratio = 0.67, cluster2: ratio = 0.67). With the following centroids and component sizes (cluster1, c = 0.28, *n* = 64, cluster2: c = 0.67, *n* = 132), cluster1 corresponded to the LOWs and cluster2 to the HIGHs, referring to the groups revealed with the k-means algorithm. For cluster1 (describing the LOWs) a significant intercept was observed (β = 0.3; SE = 0.01; *z* = 23.83; *p* < 0.001) and significant effects of the predictor subscale *Musical Training* (β = 0.04; SE = 0.01; *z* = 3.08; *p* = 0.002). For cluster2 (describing the HIGHs) a significant intercept was observed (β = 0.64; SE = 0.02; *z* = 35.44; *p* < 0.001) and significant effects of the predictor’s subscale *Musical Perception* (β = 0.06; SE = 0.02; *z* = 2.88; *p* = 0.004) and *Musical Training* (β = 0.04; SE = 0.01; *z* = 2.9; *p* = 0.021), as well as an effect of Emotional Engagement with a negative coefficient (β = −0.03; SE = 0.01; *z* = −2.1; *p* = 0.036). In order to avoid multicollinearity issues, our interpretation focuses on the PCA based analysis.

It is noteworthy that, because some participants showed PLV values that were close to the high/low synchronizer cut-off (between 0.45 and 0.55), we additionally performed a control analysis, where these participants were excluded. The analysis pipeline (PCA analysis and latent class regression models) was repeated with this sample (173 total participants; HIGHs, *n* = 101). The latent class regression models with the PCA components as predictors and with the Gold-MSI subscales as predictors both confirmed the results of our original analysis, whereas the clusters were better separated. Cluster1 exactly overlapped with the LOWs. A significant predictor was the PCA Component 5 with the highest relation to the *Musical Training* subscale (for the analysis based on the Gold-MSI subscales: only the *Musical Training* subscale showed a significant effect). Cluster 2 exactly overlapped with the HIGHs. There were significant effects of Component 1 related to *Musical Perception*, Component 5 related to *Musical Training*, and a trend for Component 4 related to *Singing Skills* (for the analysis based on the Gold-MSI subscales: next to a significant effect of the *Musical Perception* scale, there was a trend for the *Musical Training scale*).

## Discussion

Here we find that populations (HIGHs and LOWs) with previously reported differences in functional and structural connectivity between frontal speech-motor and auditory cortex (Assaneo et al., 2019a), as well as in perceptual abilities in tasks involving speech (Assaneo et al., 2021) or sound (Kern et al., 2021), also differ in self-reported musical sophistication. Musical sophistication was higher in high synchronizers compared to low synchronizers. Although in line with previous results (Assaneo et al., 2019a) high synchronizers reported more years of musical training, groups did not differ in the type of instrument they were trained on. The Gold-MSI subscales *Musical Perception* and *Musical Training* both significantly predicted speech auditory-motor coupling in the HIGHs, even when the highly correlated self-assessments of musical perception and training were disentangled by a PCA analysis. In contrast, only *Musical Training* predicted speech auditory-motor coupling in the LOWs. This provides further evidence that perception also relies on auditory-motor coupling and that speech and musical processing share certain mechanisms.

### Speech Auditory-Motor Synchronization Inferred From Musical Training and Perception

Musical training has been suggested to enhance auditory (Parbery-Clark et al., 2013) and speech perception (Parbery-Clark et al., 2011; Strait and Kraus, 2011; Swaminathan et al., 2015; Varnet et al., 2015; Zendel et al., 2015; Coffey et al., 2017a, b), through a plasticity-related increment in auditory-motor cortex coupling (Du and Zatorre, 2017). Thus, it is not surprising that the component related to musical training resulted in a good predictor of speech-to-speech synchrony (a behavioral measurement previously linked to structural connectivity between motor and auditory cortices). On the other hand, it has been argued that musical perceptual selectivity does not require formal musical training (Boebinger et al., 2021). Inherent auditory skills might shape auditory and speech perception, while formal musical training can additionally contribute (Mankel and Bidelman, 2018). We find, in line with the latter, that the components most strongly related to both the subscales *Musical Training* and *Musical Perception* (as well as a trend of the subscale *Singing Skills*) predict speech-to-speech synchrony in high synchronizers. Importantly, this was the case even when the highly correlated Gold-MSI subscores were decorrelated. Principal component analysis was used to decorrelate the Gold-MSI subscores. Because there is no one-on-one mapping between the Gold-MSI subscores and the components, an additional control analysis on the original data was used, which confirmed our interpretation.

Interestingly, components most strongly related to the subscales *Musical Training* and *Perception* contributed differently to predict auditory-motor synchronization within the HIGHs and LOWs. The higher the musical training the higher the auditory-motor synchronization (measured as PLV) in the HIGHs and LOWs, however, with a steeper increase (i.e., slope) in HIGHs. In contrast, only in the HIGHs, higher musical perception scores related to higher auditory-motor synchronization. Possibly, to become a high synchronizer musical training needs to transfer to perceptual abilities. Furthermore, perceptual abilities might have been in general low in the group of low synchronizers.

We were able to predict whether a participant was a high or a low synchronizer based on a self-assessment questionnaire (the Gold-MSI). Even though the group affiliation as high or low synchronizer could be predicted from aspects of self-reported musical sophistication, in our previous research we showed that the SSS-test correlated with auditory perception beyond effects of musical sophistication (Kern et al., 2021). This suggests that there is a relation between speech-to-speech synchronization and musical perception and training, whereas speech-to-speech synchronization (as accessed with the behavioral SSS-test) might be a more direct estimate of certain auditory perception abilities and auditory-motor cortex coupling. In summary, our findings are in line with previous research suggesting a partially overlapping mechanism of speech and music that are similarly affected by musical training (Peretz et al., 2015; Sammler and Elmer, 2020).

While future research is required to identify the brain mechanism underlying the connection between speech-to-speech synchronization and musical perception and training, we hypothesize that the structural features of the left arcuate fasciculus can, at least partially, explain our observations. This white matter pathway connects temporal auditory regions with frontal speech production areas and has been proposed to be the main pathway for the dorsal language stream (Hickok and Poeppel, 2007). This fasciculus has been shown to be enhanced in high synchronizers compared to lows (Assaneo et al., 2019a). Interestingly, research shows that brain plasticity can be modulated by musical training, especially a positive correlation between structural connectivity measures of the arcuate fasciculus and musical training has been reported (Halwani et al., 2011; Steele et al., 2013). Bringing those results together, we suggest that musical training can enhance the structural connectivity between speech perception and production regions resulting in high levels of speech-to-speech synchrony. Furthermore, the same white matter structure (i.e., left arcuate fasciculus) has been shown to be involved in auditory perception (Vaquero et al., 2021) as well as in statistical word learning (López-Barroso et al., 2013).

### Musical Sophistication of Low and High Synchronizers With Respect to a Norm Population

Interestingly, low speech auditory-motor synchronizers showed musical sophistication scores in the lower percentiles (Figure 4) of the English norm population (as reported by: Müllensiefen et al., 2014). In contrast, high synchronizers showed a similar distribution compared to the norm. Overall, our participant sample (high and low synchronizers combined) scored lower compared to the Gold-MSI English norm population. Others had previously reported lower Gold-MSI scores in a German replication of the Gold-MSI inventory compared to the English norm population (Schaal et al., 2014). When we compare our data to this German sample (which was much smaller compared to the English norm population), high synchronizers scored above this sample for all scales and low synchronizers scored below the sample at the General Sophistication and Musical Training scales (whereas Emotional Attachment was above the sample). Overall, the General Sophistication score of our sample was not significantly different from the reported German data. This suggests that the SSS-test provides an indication of “higher” vs. “lower” musical sophistication within a group of individuals of the general population.

Overall LOWs showed reduced scores compared to HIGHs in general musical sophistication and for all subscales of the Gold-MSI, but the emotional engagement scale. Surprisingly, LOWs also showed lower scores compared to HIGHs for the *Active Engagement* subscale (even though this scale wasn’t predictive of the auditory-motor synchronization strength). One could speculate that the LOWs might have some reduced capability of enjoying rhythmic and motoric aspects of music, probably due to having weaker auditory-motor coupling also in the perceptual not only the active production pathway. The lower appreciation of rhythmic aspects of music might in turn affect the motivation to learn a musical instrument and train one’s musical skills. In contrast, the emotional engagement was comparable for high and low synchronizers, suggesting that emotional engagement aspects might be dependent only to a small degree on rhythm processing capabilities. Future research is required to better understand a possible connection between active engagement and rhythmic auditory-motor coupling.

### Type of Musical Instrument Training and Speech Auditory-Motor Synchronization

Musical training might impact different aspects of auditory perception and higher cognitive processes (Merrett et al., 2013; Slater et al., 2017). For example, instruments such as percussion might particularly train rhythmic abilities and have been shown to more strongly impact speech in noise perception (Slater and Kraus, 2016; Zhang et al., 2020). Our findings, however, suggest no impact of the choice of the musical instrument on speech synchronization behavior. Individuals with high vs. low synchrony behavior—despite strong differences in whether they received musical training and the overall years of training—showed no differences in the type of musical instrument they were trained on. In line with our findings, others have shown no effect of the type of musical training on auditory psychophysical measures (comparing violinist and pianists: Carey et al., 2015), or on an age-related benefit from musical training for speech perception in noise (comparing several instrument families: Zhang et al., 2020).

A limitation of our findings is, that in contrast to previous studies, we did not investigate professional musicians. Furthermore, the type of musical training (instrument family) was not controlled in our study, so that for example very few individuals in our sample perceived percussion training (overall, *n* = 1, HIGHs, *n* = 1), making conclusions on the effect of this specific instrument family not feasible. Furthermore, our analysis is limited in that the Gold-MSI inventory accesses only the overall years of training, but not the years of musical training per instrument.

## Conclusion

Our findings show that speech-to-speech synchronization behavior can be predicted by aspects of self-reported musical sophistication such as musical training and musical perception (and singing skills). Our findings provide further evidence that auditory musical perception also relies on auditory-motor coupling and that speech and musical processing share certain mechanisms.

## Data Availability Statement

The data analyzed in this study is subject to the following licenses/restrictions: the anonymized process data will be made available. Requests to access these datasets should be directed to corresponding author JR, johanna.rimmele@ae.mpg.de.

## Ethics Statement

The experimental procedures involving testing of human participants were reviewed and approved by the Ethics Council of the Max Planck Society (No. 2017_12). The participants provided their written informed consent to participate in this study.

## Author Contributions

JR, PK, DP, and MFA designed the experiments. JR analyzed the data and wrote the manuscript. JR, KF, and MFA discussed the analysis. CL and JR illustrated the data. All authors edited the manuscript.

## Conflict of Interest

The authors declare that the research was conducted in the absence of any commercial or financial relationships that could be construed as a potential conflict of interest.

## Publisher’s Note

All claims expressed in this article are solely those of the authors and do not necessarily represent those of their affiliated organizations, or those of the publisher, the editors and the reviewers. Any product that may be evaluated in this article, or claim that may be made by its manufacturer, is not guaranteed or endorsed by the publisher.

## Footnotes

1
www.python.org

## References

[B1] Arthur D. Vassilvitskii S. (2007). “K-means++: the advantages of careful seeding,” in *Proceedings of the 18th Annual ACM-SIAM Symposium on Discrete Algorithms*, (Philadelphia, PA: Society for Industrial and Applied Mathematics).

[B2] Assaneo M. F. Orpella J. Ripollés P. Noejovich L. López-Barroso D. de Diego-Balaguer R. (2020). Population-level differences in the neural substrates supporting statistical learning. *bioRxiv* [Preprint]. 10.1101/2020.07.03.187260 bioRxiv:2020.07.03.187260, PMC9292101 35793349

[B3] Assaneo M. F. Poeppel D. (2018). The coupling between auditory and motor cortices is rate-restricted: evidence for an intrinsic speech-motor rhythm. *Sci. Adv.* 4:eaao3842. 10.1126/sciadv.aao3842 29441362 PMC5810610

[B4] Assaneo M. F. Ripollés P. Orpella J. Lin W. M. de Diego-Balaguer R. Poeppel D. (2019a). Spontaneous synchronization to speech reveals neural mechanisms facilitating language learning. *Nat. Neurosci.* 22 627–632. 10.1038/s41593-019-0353-z 30833700 PMC6435400

[B5] Assaneo M. F. Rimmele J. M. Orpella J. Ripollés P. de Diego-Balaguer R. Poeppel D. (2019b). The lateralization of speech-brain coupling is differentially modulated by intrinsic auditory and top-down mechanisms. *Front. Integr. Neurosci.* 13:28. 10.3389/fnint.2019.00028 31379527 PMC6650591

[B6] Assaneo M. F. Rimmele J. M. Sanz Perl Y. Poeppel D. (2021). Speaking rhythmically can shape hearing. *Nat. Hum. Behav.* 5 71–82. 10.1038/s41562-020-00962-0 33046860

[B7] Bailey J. A. Zatorre R. J. Penhune V. B. (2014). Early musical training is linked to gray matter structure in the ventral premotor cortex and auditory-motor rhythm synchronization performance. *J. Cogn. Neurosci.* 26 755–767. 10.1162/jocn_a_00527 24236696

[B8] Boebinger D. Evans S. Rosen S. Lima C. F. Manly T. Scott S. K. (2015). Musicians and non-musicians are equally adept at perceiving masked speech. *J. Acoust. Soc. Am.* 137 378–387. 10.1121/1.4904537 25618067 PMC4434218

[B9] Boebinger D. Norman-Haignere S. V. McDermott J. H. Kanwisher N. (2021). Music-selective neural populations arise without musical training. *J. Neurophysiol.* 125 2237–2263. 10.1152/jn.00588.2020 33596723 PMC8285655

[B10] Carey D. Rosen S. Krishnan S. Pearce M. T. Shepherd A. Aydelott J. (2015). Generality and specificity in the effects of musical expertise on perception and cognition. *Cognition* 137 81–105. 10.1016/j.cognition.2014.12.005 25618010

[B11] Chen X. Affourtit J. Ryskin R. Regev T. I. Norman-Haignere S. Jouravlev O. (2021). The human language system does not support music processing. *bioRxiv* [Preprint]. 10.1101/2021.06.01.446439 bioRxiv:2021.06.01.446439, PMC10505454 37005063

[B12] Coffey E. B. J. Chepesiuk A. M. P. Herholz S. C. Baillet S. Zatorre R. J. (2017a). Neural correlates of early sound encoding and their relationship to speech-in-noise perception. *Front. Neurosci.* 11:479. 10.3389/fnins.2017.00479 28890684 PMC5575455

[B13] Coffey E. B. J. Mogilever N. B. Zatorre R. J. (2017b). Speech-in-noise perception in musicians: a review. *Hear. Res.* 352 49–69. 10.1016/j.heares.2017.02.006 28213134

[B14] Di Liberto G. M. Pelofi C. Shamma S. de Cheveigné A. (2020). Musical expertise enhances the cortical tracking of the acoustic envelope during naturalistic music listening. *Acoust. Sci. Technol.* 41 361–364. 10.1250/ast.41.361

[B15] Doelling K. B. Poeppel D. (2015). Cortical entrainment to music and its modulation by expertise. *Proc. Natl. Acad. Sci. U.S.A.* 112 E6233–E6242. 10.1073/pnas.1508431112 26504238 PMC4653203

[B16] Du Y. Zatorre R. J. (2017). Musical training sharpens and bonds ears and tongue to hear speech better. *Proc. Natl. Acad. Sci. U.S.A.* 114 13579–13584. 10.1073/pnas.1712223114 29203648 PMC5754781

[B17] Fiedler D. Müllensiefen D. (2015). Validation of the Gold-MSI questionnaire to measure musical sophistication of german students at secondary education schools. *Res. Music Educ.* 36 199–219.

[B18] Ghitza O. (2011). Linking speech perception and neurophysiology: speech decoding guided by cascaded oscillators locked to the input rhythm. *Front. Psychol.* 2:130. 10.3389/fpsyg.2011.00130 21743809 PMC3127251

[B19] Giraud A.-L. Poeppel D. (2012). Cortical oscillations and speech processing: emerging computational principles and operations. *Nat. Neurosci.* 15 511–517. 22426255 10.1038/nn.3063 PMC4461038

[B20] Gross J. Hoogenboom N. Thut G. Schyns P. Panzeri S. Belin P. (2013). Speech rhythms and multiplexed oscillatory sensory coding in the human brain. *PLoS Biol.* 11:e1001752. 10.1371/journal.pbio.1001752 24391472 PMC3876971

[B21] Guo Z. Wu X. Li W. Jones J. A. Yan N. Sheft S. (2017). Top-down modulation of auditory-motor integration during speech production: the role of working memory. *J. Neurosci.* 37:10323. 10.1523/JNEUROSCI.1329-17.2017 28951450 PMC6596622

[B22] Halwani G. F. Loui P. Ruber T. Schlaug G. (2011). Effects of practice and experience on the arcuate fasciculus: comparing singers, instrumentalists, and non-musicians. *Front. Psychol.* 2:156. 10.3389/fpsyg.2011.00156 21779271 PMC3133864

[B23] Harding E. E. Sammler D. Henry M. J. Large E. W. Kotz S. A. (2019). Cortical tracking of rhythm in music and speech. *Neuroimage* 185 96–101. 10.1016/j.neuroimage.2018.10.037 30336253

[B24] Hartigan J. A. Hartigan P. M. (1985). The dip test of unimodality. *Ann. Stat.* 13 70–84. 10.1214/aos/1176346577

[B25] Hickok G. Poeppel D. (2007). The cortical organization of speech processing. *Nat. Rev. Neurosci.* 8 393–402. 10.1038/nrn2113 17431404

[B26] Hunter J. D. (2007). Matplotlib: a 2D graphics environment. *Comput. Sci. Eng.* 9, 90–95. 10.1109/MCSE.2007.55

[B27] Hutchins S. Larrouy-Maestri P. Peretz I. (2014). Singing ability is rooted in vocal-motor control of pitch. *Atten. Percept. Psychophys.* 76 2522–2530. 10.3758/s13414-014-0732-1 25060548

[B28] Hyafil A. Fontolan L. Kabdebon C. Gutkin B. Giraud A.-L. (2015). Speech encoding by coupled cortical theta and gamma oscillations. *Elife* 4:e06213. 10.7554/eLife.06213 26023831 PMC4480273

[B29] Keitel A. Gross J. Kayser C. (2018). Perceptually relevant speech tracking in auditory and motor cortex reflects distinct linguistic features. *PLoS Biol.* 16:e2004473. 10.1371/journal.pbio.2004473 29529019 PMC5864086

[B30] Kern P. Assaneo M. F. Endres D. Poeppel D. Rimmele J. M. (2021). Preferred auditory temporal processing regimes and auditory-motor synchronization. *Psychon. Bull. Rev.* 10.3758/s13423-021-01933-w [Epub ahead of print]. 34100222 PMC8642338

[B31] Kösem A. Bosker H. R. Takashima A. Meyer A. Jensen O. Hagoort P. (2018). Neural entrainment determines the words we hear. *Curr. Biol* 28 2867-2875.e3. 10.1016/j.cub.2018.07.023 30197083

[B32] Kösem A. van Wassenhove V. (2017). Distinct contributions of low- and high-frequency neural oscillations to speech comprehension. *Lang. Cogn. Neurosci.* 32 536–544. 10.1080/23273798.2016.1238495

[B33] Lachaux J.-P. Rodriguez E. Martinerie J. Varela F. J. (1999). Measuring phase synchrony in brain signals. *Hum. Brain Mapp.* 8 194–208. 10.1002/(sici)1097-0193(1999)8:4<194::aid-hbm4>3.0.co;2-c 10619414 PMC6873296

[B34] Leisch F. (2004). FlexMix: a general framework for finite mixture models and latent class regression in R. *J. Stat. Softw.* 11 1–18.

[B35] Levitin D. J. (2012). What does it mean to be musical? *Neuron* 73 633–637. 10.1016/j.neuron.2012.01.017 22365540

[B36] López-Barroso D. Catani M. Ripollés P. Dell’Acqua F. Rodríguez-Fornells A. de Diego-Balaguer R. (2013). Word learning is mediated by the left arcuate fasciculus. *Proc. Natl. Acad. Sci. U.S.A.* 110 13168–13173. 10.1073/pnas.1301696110 23884655 PMC3740909

[B37] Luo H. Poeppel D. (2007). Phase patterns of neuronal responses reliably discriminate speech in human auditory cortex. *Neuron* 54 1001–1010. 10.1016/j.neuron.2007.06.004 17582338 PMC2703451

[B38] Mankel K. Bidelman G. M. (2018). Inherent auditory skills rather than formal music training shape the neural encoding of speech. *Proc. Natl. Acad. Sci.U.S.A.* 115:13129. 10.1073/pnas.1811793115 30509989 PMC6304957

[B39] Merrett D. L. Peretz I. Wilson S. J. (2013). Moderating variables of music training-induced neuroplasticity: a review and discussion. *Front. Psychol.* 4:606. 10.3389/fpsyg.2013.00606 24058353 PMC3766835

[B40] Moreno S. Bidelman G. M. (2014). Examining neural plasticity and cognitive benefit through the unique lens of musical training. *Music Window Hear. Brain* 308 84–97. 10.1016/j.heares.2013.09.012 24079993

[B41] Müllensiefen D. Gingras B. Musil J. Stewart L. (2014). The musicality of non-musicians: an index for assessing musical sophistication in the general population. *PLoS One* 9:e89642. 10.1371/journal.pone.0089642 24586929 PMC3935919

[B42] Norman-Haignere S. Kanwisher N. G. McDermott J. H. (2015). Distinct cortical pathways for music and speech revealed by hypothesis-free voxel decomposition. *Neuron* 88 1281–1296. 10.1016/j.neuron.2015.11.035 26687225 PMC4740977

[B43] Parbery-Clark A. Strait D. L. Anderson S. Hittner E. Kraus N. (2011). Musical experience and the aging auditory system: implications for cognitive abilities and hearing speech in noise. *PLoS One* 6:e18082. 10.1371/journal.pone.0018082 21589653 PMC3092743

[B44] Parbery-Clark A. Strait D. L. Hittner E. Kraus N. (2013). Musical training enhances neural processing of binaural sounds. *J. Neurosci.* 33 16741. 10.1523/JNEUROSCI.5700-12.2013 24133275 PMC6618537

[B45] Pelofi C. de Gardelle V. Egré P. Pressnitzer D. (2017). Interindividual variability in auditory scene analysis revealed by confidence judgements. *Philos. Trans. R. Soc. B Biol. Sci.* 372:20160107. 10.1098/rstb.2016.0107 28044018 PMC5206275

[B46] Peretz I. Vuvan D. Lagrois M. -É Armony J. L. (2015). Neural overlap in processing music and speech. *Philos. Trans. R. Soc. B Biol. Sci.* 370:20140090. 10.1098/rstb.2014.0090 25646513 PMC4321131

[B47] Puschmann S. Baillet S. Zatorre R. J. (2018). Musicians at the cocktail party: neural substrates of musical training during selective listening in multispeaker situations. *Cereb. Cortex* 29 3253–3265. 10.1093/cercor/bhy193 PMC6644853 30137239

[B48] Puschmann S. Regev M. Baillet S. Zatorre R. J. (2021). MEG intersubject phase locking of stimulus-driven activity during naturalistic speech listening correlates with musical training. *J. Neurosci.* 41:2713. 10.1523/JNEUROSCI.0932-20.2020 33536196 PMC8018743

[B49] Rimmele J. M. Morillon B. Poeppel D. Arnal L. H. (2018). proactive sensing of periodic and aperiodic auditory patterns. *Trends Cogn. Sci.* 22 870–882. 10.1016/j.tics.2018.08.003 30266147

[B50] Rimmele J. M. Poeppel D. Ghitza O. (2021). Acoustically driven cortical delta oscillations underpin prosodic chunking. *eNeuro* 8:ENEURO.0562-20.2021. 10.1523/ENEURO.0562-20.2021 34083380 PMC8272402

[B51] Rimmele J. M. Zion Golumbic E. Schröger E. Poeppel D. (2015). The effects of selective attention and speech acoustics on neural speech-tracking in a multi-talker scene. *Cortex* 68 144–154. 10.1016/j.cortex.2014.12.014 25650107 PMC4475476

[B52] Ruggles D. R. Freyman R. L. Oxenham A. J. (2014). Influence of musical training on understanding voiced and whispered speech in noise. *PLoS One* 9:e86980. 10.1371/journal.pone.0086980 24489819 PMC3904968

[B53] Sammler D. (2020). Splitting speech and music. *Science* 367:974. 10.1126/science.aba7913 32108099

[B54] Sammler D. Elmer S. (2020). Advances in the neurocognition of music and language. *Brain Sci.* 10:509. 10.3390/brainsci10080509 32748810 PMC7464495

[B55] Schaal N. K. Bauer A.-K. R. Müllensiefen D. (2014). Der Gold-MSI: replikation und validierung eines fragebogeninstrumentes zur messung musikalischer erfahrenheit anhand einer deutschen stichprobe. *Music Sci.* 18 423–447. 10.1177/1029864914541851

[B56] Slater J. Ashley R. Tierney A. Kraus N. (2018). Got rhythm? Better inhibitory control is linked with more consistent drumming and enhanced neural tracking of the musical beat in adult percussionists and nonpercussionists. *J. Cogn. Neurosci.* 30 14–24. 10.1162/jocn_a_01189 28949825

[B57] Slater J. Azem A. Nicol T. Swedenborg B. Kraus N. (2017). Variations on the theme of musical expertise: cognitive and sensory processing in percussionists, vocalists and non-musicians. *Eur. J. Neurosci.* 45 952–963. 10.1111/ejn.13535 28177157 PMC5378620

[B58] Slater J. Kraus N. (2016). The role of rhythm in perceiving speech in noise: a comparison of percussionists, vocalists and non-musicians. *Cogn. Process* 17 79–87. 10.1007/s10339-015-0740-7 26445880 PMC5019948

[B59] Smith Z. M. Delgutte B. Oxenham A. J. (2002). Chimaeric sounds reveal dichotomies in auditory perception. *Nature* 416 87–90. 11882898 10.1038/416087a PMC2268248

[B60] Steele C. J. Bailey J. A. Zatorre R. J. Penhune V. B. (2013). Early musical training and white-matter plasticity in the corpus callosum: evidence for a sensitive period. *J. Neurosci.* 33:1282. 10.1523/JNEUROSCI.3578-12.2013 23325263 PMC6704889

[B61] Strait D. L. Kraus N. (2011). Can you hear me now? Musical training shapes functional brain networks for selective auditory attention and hearing speech in noise. *Front. Psychol.* 2:113. 10.3389/fpsyg.2011.00113 21716636 PMC3115514

[B62] Swaminathan J. Mason C. R. Streeter T. M. Best V. Kidd G. J. Patel A. D. (2015). Musical training, individual differences and the cocktail party problem. *Sci. Rep.* 5:11628. 10.1038/srep11628 26112910 PMC4481518

[B63] Tal I. Large E. W. Rabinovitch E. Wei Y. Schroeder C. E. Poeppel D. (2017). Neural entrainment to the beat: the “missing-pulse”. *Phenomenon. J. Neurosci.* 37:6331. 10.1523/JNEUROSCI.2500-16.2017 28559379 PMC5490067

[B64] Vaquero L. Ramos-Escobar N. Cucurell D. François C. Putkinen V. Segura E. (2021). Arcuate fasciculus architecture is associated with individual differences in pre-attentive detection of unpredicted music changes. *Neuroimage* 229:117759. 10.1016/j.neuroimage.2021.117759 33454403

[B65] Varnet L. Wang T. Peter C. Meunier F. Hoen M. (2015). How musical expertise shapes speech perception: evidence from auditory classification images. *Sci. Rep.* 5:14489. 10.1038/srep14489 26399909 PMC4585866

[B66] Waskom M. (2021). Seaborn: statistical data visualization. *J. Open Source Softw.* 6:3021. 10.21105/joss.03021

[B67] Wang K. Shamma S. A. (1994). Modeling the auditory functions in the primary cortex. *Opt. Eng.* 33 2143–2150. 10.1117/12.172243

[B68] Yang X. Wang K. Shamma S. A. (1992). Auditory representations of acoustic signals. *IEEE Trans. Inf. Theor.* 38 824–839. 10.1109/18.119739

[B69] Yoo J. Bidelman G. M. (2019). Linguistic, perceptual, and cognitive factors underlying musicians’ benefits in noise-degraded speech perception. *Hear. Res.* 377 189–195. 10.1016/j.heares.2019.03.021 30978607 PMC6511496

[B70] Zatorre R. J. (2005). Music, the food of neuroscience? *Nature* 434 312–315. 10.1038/434312a 15772648

[B71] Zatorre R. J. Chen J. L. Penhune V. B. (2007). When the brain plays music: auditory–motor interactions in music perception and production. *Nat. Rev. Neurosci.* 8 547–558. 10.1038/nrn2152 17585307

[B72] Zendel B. R. Tremblay C.-D. Belleville S. Peretz I. (2015). The impact of musicianship on the cortical mechanisms related to separating speech from background noise. *J. Cogn. Neurosci.* 27 1044–1059. 10.1162/jocn_a_00758 25390195

[B73] Zhang L. Fu X. Luo D. Xing L. Du Y. (2020). Musical experience offsets age-related decline in understanding speech-in-noise: type of training does not matter, working memory is the key. *Ear Hear.* 42 258–270. 10.1097/AUD.0000000000000921 32826504 PMC7969154

